# Successful Use of Octreotide for Refractory Postural Hypotension in a Patient With Parkinson’s Disease and Heart Failure With Preserved Ejection Fraction

**DOI:** 10.7759/cureus.92650

**Published:** 2025-09-18

**Authors:** Kyaw Thet, Daniel Duric

**Affiliations:** 1 Internal Medicine/Geriatrics, Aneurin Bevan University Health Board, Abergavenny, GBR; 2 Geriatrics, Aneurin Bevan University Health Board, Abergavenny, GBR

**Keywords:** autonomic nervous system dysfunction, heart failure with preserved ejection fraction, parkinson's disease non-motor symptoms, parkinson’s management, postural orthostatic hypotension

## Abstract

Postural hypotension, particularly in patients with autonomic dysfunction secondary to Parkinson’s disease, presents a complex therapeutic challenge, especially when standard treatments fail. We report the case of an elderly patient admitted to a UK district general hospital with worsening diarrhoea and profound postural hypotension. The patient’s background included Parkinson’s disease, heart failure with preserved ejection fraction (HFpEF), ulcerative colitis, type 2 diabetes mellitus, and other chronic comorbidities. Initial management involved standard pharmacological therapy with fludrocortisone and midodrine, titrated to maximum tolerated doses. Despite this, the patient remained significantly symptomatic, unable to sit upright or stand. Repeated lying and standing blood pressure measurements confirmed substantial postural drops, and physiotherapy input was limited by hemodynamic instability. Given the ongoing symptoms and potential risks of fluid overload in the setting of HFpEF, fludrocortisone was discontinued. Following local governance approval through the NHS trust’s health board, required due to the unlicensed nature of octreotide for this indication, a stepwise titration of subcutaneous octreotide infusion was initiated. The patient exhibited progressive improvement at each stage, ultimately regaining the ability to sit and stand without orthostatic symptoms. Blood pressure readings stabilised in parallel with clinical recovery. This case highlights the potential utility of octreotide for managing refractory postural hypotension in the NHS, where standard treatments may be ineffective or contraindicated, and demonstrates the value of structured trust-level governance for safe off-label prescribing.

## Introduction

Postural hypotension, defined as a sustained drop in systolic blood pressure of ≥20 mmHg or diastolic pressure of ≥10 mmHg within three minutes of standing, is a common and disabling condition, particularly in older adults and those with autonomic dysfunction [1]. It is frequently encountered in patients with Parkinson’s disease due to neurogenic causes, where degeneration of sympathetic vasomotor pathways impairs vascular tone and baroreflex responses.

In the UK, standard management typically includes non-pharmacological measures (e.g., compression stockings, salt/fluid intake, head-up tilt) and pharmacological agents such as midodrine and fludrocortisone, both of which are available on NHS formularies and recommended in guidance for neurogenic orthostatic hypotension [1]. However, a subset of patients remains symptomatic despite maximal dosing, particularly those with multiple comorbidities such as heart failure, diabetes mellitus, and autonomic neuropathy.

Octreotide, a somatostatin analogue, has been proposed as a second-line agent in treatment-resistant cases due to its ability to induce splanchnic vasoconstriction and reduce venous pooling. However, its use in this context remains off-label in the UK and is not included in national guidance, largely due to a lack of large-scale clinical trials and its corresponding unlicensed status for this indication. Consequently, it is not routinely included in NHS protocols for postural hypotension, and prescribing requires local governance approval, often on a case-by-case basis via trust managers. We present the case of a frail, multimorbid patient with Parkinson’s disease, ulcerative colitis, and heart failure with preserved ejection fraction, who demonstrated significant symptomatic and hemodynamic improvement following the stepwise introduction of octreotide after failing conventional therapies.

## Case presentation

An elderly patient with a complex background of idiopathic Parkinson’s disease, heart failure with preserved ejection fraction (HFpEF), type 2 diabetes mellitus, and ulcerative colitis was admitted to a UK district general hospital on 3 June 2024 with worsening diarrhoea. At baseline, the patient lived alone and was independent in personal care, mobilising with a Zimmer frame. On presentation, they reported diarrhoea for four weeks, occurring seven to eight times daily, without vomiting, melaena, or gastrointestinal bleeding.

During admission, the patient also developed marked postural hypotension, with lying-to-standing systolic blood pressure drops exceeding 30 mmHg on repeated measurements. The hypotension significantly limited their ability to sit upright or participate in physiotherapy and was associated with pre-syncopal symptoms. Initial management included midodrine, titrated to 10 mg three times daily, and fludrocortisone, titrated to 200 µg once daily. Despite several weeks of maximum tolerated dosing, there was no meaningful improvement in symptoms or hemodynamic response. Given the patient’s comorbid HFpEF, fludrocortisone was discontinued in early October due to concerns regarding volume overload and fluid retention.

Following continued symptomatic instability, a formal request was submitted to the local NHS health board for off-label use of subcutaneous octreotide, which is not licensed in the UK for the treatment of postural hypotension. Approval was granted on a case-by-case basis via trust governance. Octreotide infusion was initiated on 24 October 2024 at a dose of 200 µg per 24 hours, delivered via a continuous subcutaneous syringe driver. The dose was increased incrementally by 50 µg each week, reaching a final dose of 400 µg per 24 hours, which was well tolerated. With each increase, the patient experienced progressive improvement in postural symptoms and blood pressure stability. By the time the target dose was reached, the patient was able to sit, stand, and mobilise short distances without presyncope.

**Figure 1:**
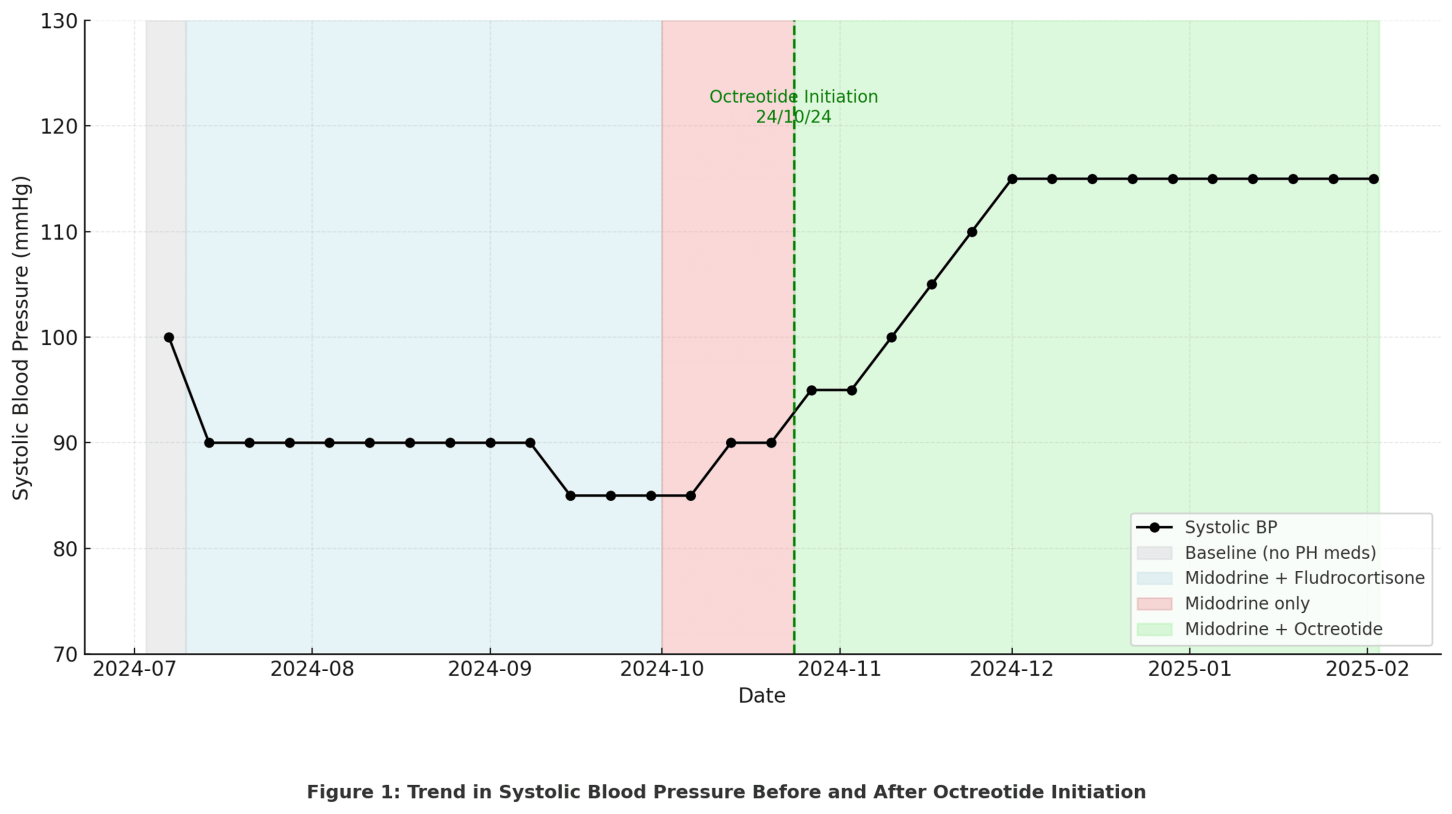
Trend in Systolic Blood Pressure Before and After Octreotide

Figure 1 illustrates the patient’s systolic blood pressure trajectory over the course of admission. From July to early October, despite treatment with midodrine (10 mg TDS (to be taken three times daily)) and fludrocortisone (200 µg daily), there was a progressive decline in average systolic blood pressure, with values falling to around 85-90 mmHg and marked postural drops exceeding 30 mmHg. Fludrocortisone was discontinued in early October due to concerns regarding fluid retention in the context of HFpEF. During this short period on midodrine monotherapy, the patient’s blood pressure remained low and symptomatic.

Following governance approval, subcutaneous octreotide infusion was initiated on 24 October 2024 at 200 µg/24 h and titrated weekly in 50 µg increments up to 400 µg/24 h. With each increase, the figure demonstrates a stepwise rise in systolic blood pressure: from ~90 mmHg pre-octreotide, to 95 mmHg at 200 µg, 100 mmHg at 250 µg, 105 mmHg at 300 µg, and 110-115 mmHg once the target dose of 400 µg/24 h was reached.

Table 1 summarises the patient’s lying and standing blood pressure measurements at key stages of treatment. At baseline (3 July 2024), there was a systolic drop of 30 mmHg on standing. Despite maximal midodrine (10 mg TDS) and fludrocortisone (200 µg daily), the patient continued to experience significant orthostatic hypotension, with lying-standing differences of ~30 mmHg persisting through September. Following discontinuation of fludrocortisone in early October, blood pressures remained low on midodrine monotherapy. After the initiation of subcutaneous octreotide infusion on 24 October 2024 (200 µg/24 h) and subsequent weekly titration up to 400 µg/24 h by early December, there was a progressive rise in standing systolic pressure (from 90 mmHg at initiation to 115-120 mmHg at discharge) with a corresponding reduction in the postural drop from 30 mmHg to 15 mmHg. No adverse effects attributable to octreotide, such as gastrointestinal symptoms or hyperglycaemia, were observed during the three-month inpatient period, including the two months spent on the maximum 400 µg/24 h dose.

**Table 1:** Lying and Standing Blood Pressure Measurements at Key Treatment Stages

Date/Stage	Lying BP (mmHg)	Standing BP (mmHg)	Postural drop (systolic mmHg)
Baseline (3 July 2024)	130/70	100/60	30
Midodrine + fludrocortisone (July-September, peak dose)	120/70	90/55	30
Midodrine only (early October, after fludrocortisone stopped)	115/70	85/50	30
Octreotide 200 µg/24 h (24 October 2024)	120/72	90/60	30
Octreotide 250 µg/24h (31 October 2024)	120/74	95/65	25
Octreotide 300 µg/24 h (7 November 2024)	128/75	108/68	20
Octreotide 350 µg/24 h (14 November 2024)	130/76	113/70	17
Octreotide 400 µg/24 h (early December 2024)	132/78	115/72	17
Discharge (3/ February 2025, on 400 µg/24 h)	135/78	120/72	15

This sustained improvement in upright systolic blood pressure correlated closely with the patient’s clinical progress: they regained the ability to sit upright, mobilise short distances, and participate in physiotherapy without presyncopal symptoms. The figure and the table therefore highlight both the failure of standard agents (midodrine and fludrocortisone) and the progressive haemodynamic benefit following octreotide initiation and titration.

The patient was discharged on 3 February 2025 with stable haemodynamics and a comprehensive medication plan, including octreotide 400 µg per 24 hours, midodrine 10 mg TDS, mesalazine (both oral and rectal formulations), prednisolone, and other agents for long-term management of Parkinson’s disease, diabetes, and ulcerative colitis. Ongoing community-based vedolizumab infusions were arranged, along with outpatient urology follow-up for long-term catheter care.

## Discussion

Postural hypotension (PH) is a frequent manifestation of autonomic dysfunction, particularly in patients with Parkinson’s disease. It significantly impairs functional capacity, increases fall risk, and contributes to hospitalisation and reduced quality of life. PH is defined as a sustained drop in systolic blood pressure of ≥20 mmHg or diastolic pressure of ≥10 mmHg within three minutes of standing, in accordance with UK clinical definitions [2]. In Parkinson’s disease, PH is typically neurogenic in origin, resulting from failure of sympathetic vasoconstriction, baroreceptor dysfunction, and impaired noradrenaline release [2].

Within the NHS, current management of neurogenic PH follows a stepwise approach, beginning with non-pharmacological strategies including increased salt and fluid intake, abdominal binders, head-up tilt during sleep, and physical counter-manoeuvres [2]. If symptoms persist, pharmacological treatment is initiated. National Institute for Health and Care Excellence (NICE) guidance recommends midodrine as the first-line agent for symptomatic neurogenic hypotension in Parkinson’s disease [1], and this is supported by *BMJ Best Practice* [2].

Midodrine is a direct-acting α1-adrenergic agonist that increases arterial and venous tone, with multiple studies confirming its efficacy in raising standing blood pressure and improving symptoms. In a landmark placebo-controlled trial, midodrine demonstrated significant improvement in orthostatic BP and symptom control in neurogenic hypotension [3]. In this patient, midodrine was titrated to 10 mg three times daily, the recommended maximum dose, with no clinical benefit.

Fludrocortisone, though not recommended by NICE, is widely used across NHS trusts and included in *BMJ Best Practice* [2]. It acts via volume expansion through sodium and water retention. However, it may be poorly tolerated in patients with pre-existing heart failure with preserved ejection fraction (HFpEF), as was the case here, due to the risk of precipitating fluid overload and exacerbating cardiac symptoms. The British National Formulary (BNF) lists fludrocortisone under “use with caution” in patients with cardiovascular disease. In our patient, fludrocortisone was trialled at 200 µg daily, but given the lack of response and background HFpEF, it was discontinued.

With persistent orthostatic symptoms despite maximal conventional therapy, and significant postural systolic drops, the team considered off-label use of octreotide. Octreotide is a somatostatin analogue that exerts its hemodynamic effect by causing splanchnic vasoconstriction, which redistributes blood centrally and reduces postural venous pooling [4]. Although not licensed for this indication in the UK [1], its mechanism of action is well-understood and has been explored in small clinical studies.

Following trust-level governance approval, octreotide was initiated at 200 µg/24 hours via subcutaneous syringe driver and titrated weekly in 50 µg increments to a final dose of 400 µg/24 hours. The patient responded progressively at each stage of titration, eventually achieving complete resolution of postural symptoms and the ability to sit and stand without dizziness. This was supported by BP observations, which showed narrowing of postural BP drop and improved upright systolic pressures.

Evidence from Hoeldtke et al. demonstrated that octreotide improved standing blood pressure and reduced presyncopal symptoms in patients with autonomic neuropathy [5]. Similarly, the 2018 consensus statement on neurogenic orthostatic hypotension recognised octreotide as a reasonable second-line option for patients unresponsive to midodrine or fludrocortisone, although it emphasises the need for cautious off-label use [6]. *BMJ Best Practice* also references octreotide as a consideration in refractory cases, though typically in specialist settings [2].

This case reinforces two important clinical messages. First, off-label prescribing within the NHS can be safe and justified when supported by local governance processes and used selectively in patients with clear unmet needs. Second, octreotide can be an effective therapy in highly selected cases of refractory postural hypotension when conventional agents fail [3,5]. While routine use cannot be recommended, this case adds to the growing body of UK-based real-world evidence supporting octreotide as a rescue agent in severe autonomic dysfunction.

## Conclusions

This case highlights the potential role of octreotide as a therapeutic option for refractory postural hypotension in patients with neurogenic autonomic dysfunction, particularly when standard NHS-approved treatments such as midodrine and fludrocortisone fail. The patient’s improvement, both symptomatically and physiologically, following stepwise titration of subcutaneous octreotide infusion illustrates its clinical value in complex, multimorbid patients. Although octreotide is not licensed for this indication in the UK, this case demonstrates that, with appropriate governance oversight and trust-level approval, off-label use can be justified and effective within the NHS framework. Further research is warranted to evaluate its broader application in this patient population.
